# AlGaInP light-emitting diodes with SACNTs as current-spreading layer

**DOI:** 10.1186/1556-276X-9-171

**Published:** 2014-04-08

**Authors:** Xia Guo, Chun Wei Guo, Yuan Hao Jin, Yu Chen, Qun Qing Li, Shou Shan Fan

**Affiliations:** 1 School of Electrical Information and Control Engineering, Beijing University of Technology, Beijing 100124, China; 2 Department of Physics and Tsinghua-Foxconn Nanotechnology Research Center, Tsinghua University, Beijing 100084, China

**Keywords:** AlGaInP, Light-emitting diodes (LEDs), Super-aligned carbon nanotube (SACNTs), Current-spreading

## Abstract

Transparent conductive current-spreading layer is important for quantum efficiency and thermal performance of light-emitting diodes (LEDs). The increasing demand for tin-doped indium oxide (ITO) caused the price to greatly increase. Super-aligned carbon nanotubes (SACNTs) and Au-coated SACNTs as current-spreading layer were applied on AlGaInP LEDs. The LEDs with Au-coated SACNTs showed good current spreading effect. The voltage bias at 20 mA dropped about 0.15 V, and the optical power increased about 10% compared with the LEDs without SACNTs.

## Background

The current-spreading effect is one of the most important factors limiting the external quantum efficiency of light-emitting diodes (LEDs) [1,2]. Limited by the mobility and thickness of the current-spreading layer, most carriers crowd under the electrode, which resulted in most photons from radiation recombination being blocked or absorbed by opaque electrode and large joule heating under the electrode [3,4]. Indium tin oxide (ITO) was widely used in case of visible wavelength LEDs as the current-spreading layer owing to its good physical properties of high optical transmittance and low sheet resistance [5]. However, due to the scarcity of the element indium on earth and consequently the soaring prices, the advantages in nanomaterials were recently investigated for the current-spreading layer, such as graphene, metal nanowires, and carbon nanotubes (CNTs) [6-8].

Graphene has high mobility and high optical transmittance [9]. However, large work function of graphene caused the large turn-on voltage with inefficient current spreading, which resulted in light emission occurring only near the p-metal regions, especially on p-GaN due to high sheet and contact resistance [10]. Also, the obvious degradation of graphene layer under 20 mW of input power restricted its actual application [11]. Ag nanowire is the strong competitor of graphene due to its intrinsically high conductivity and favorable optical transparency. However, except for the easy oxidation at ambient environment, the electromigration of silver ions under bias could pose a long-term stability issue [12].

Recently, the optical output power of LEDs was first improved by using the combination of graphene film and Ag nanowires as current-spreading layer. The sheet resistance decreases from 500 Ω of bare graphene to about 30 Ω because the silver nanowires bridged the grain boundaries of graphene and increased the conduction channels [13].

Among these three nanomaterials, CNTs have the most mature fabrication technology. In this work, AlGaInP LEDs with CNTs only and 2-nm-thick Au-coated CNTs as current-spreading layers were fabricated. The LEDs with Au-coated CNTs showed good current spreading effect.

## Methods

The AlGaInP LEDs were grown on n-GaAs substrate by metal-organic chemical vapor deposition. Fifteen pairs of Al_0.6_Ga_0.4_As/AlAs with distributed Bragg reflectors (DBRs) were grown on 100-nm-thick GaAs buffer layer. The active region was composed of 800-nm-thick 60-period (Al_0.5_Ga_0.5_)_0.5_In_0.5_P/(Al_0.1_Ga_0.9_)_0.5_In_0.5_P multiquantum wells, which were sandwiched in p- and n-(Al_0.7_Ga_0.3_)_0.5_In_0.5_P cladding layer for electron and hole confinement. In order to study the current-spreading effect of CNTs, only 500-nm-thick Mg-doped p-GaP window layer with the doping density of 5 × 10^18^ cm^−3^ was grown on top.

**Figure 1:**
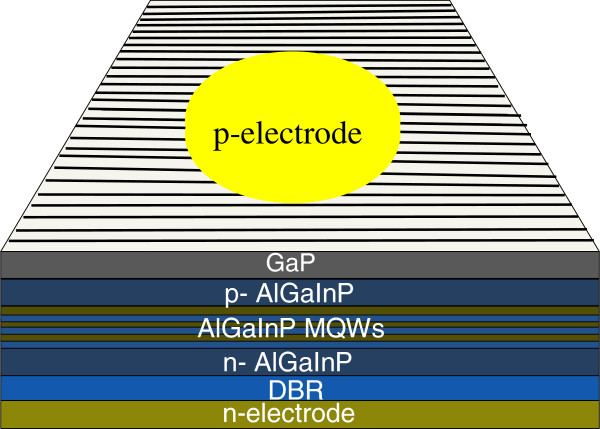
**Schematic diagram of fabricated (Al**_**0.5**_**Ga**_**0.5**_**)**_**0.5**_**In**_**0.5**_**P/(Al**_**0.1**_**Ga**_**0.9**_**)**_**0.5**_**In**_**0.5**_**P MQWs LED structure with SACNT or Au-coated CNT as current-spreading layer.**

The 50/150/200-nm-thick Au/BeAu/Au with 100-μm diameter was first deposited and then patterned by wet etching as a p-type electrode. A super-aligned CNT (SACNT) film is drawn continuously from multiwalled CNT arrays [14]. To improve the conductivity of the as-drawn SACNT films, 2-nm-thick Au was further coated on the SACNTs by magnetron sputtering methods [15]. Then the SACNT thin film was put and stuck on the surface of the LED wafer by Van der Waals force. In order to keep the tubes in place, additional 150/300-nm-thick Ti/Au was deposited and patterned on the p-type electrode. The street was exposed in the inductively coupled plasma etcher using O_2_ gas in order to avoid the current leakage through the tubes after wafer dicing process. The AuGeNi/Au metals as n-electrode were deposited on the n-GaAs substrate by sputtering. For comparison, bare AlGaInP LEDs without SACNT current-spreading layer were fabricated at the same time. A schematic diagram of LEDs in this experiment is shown in Figure 1. The chip size was 300 μm × 300 μm in this work.

## Results and discussion

**Figure 2:**
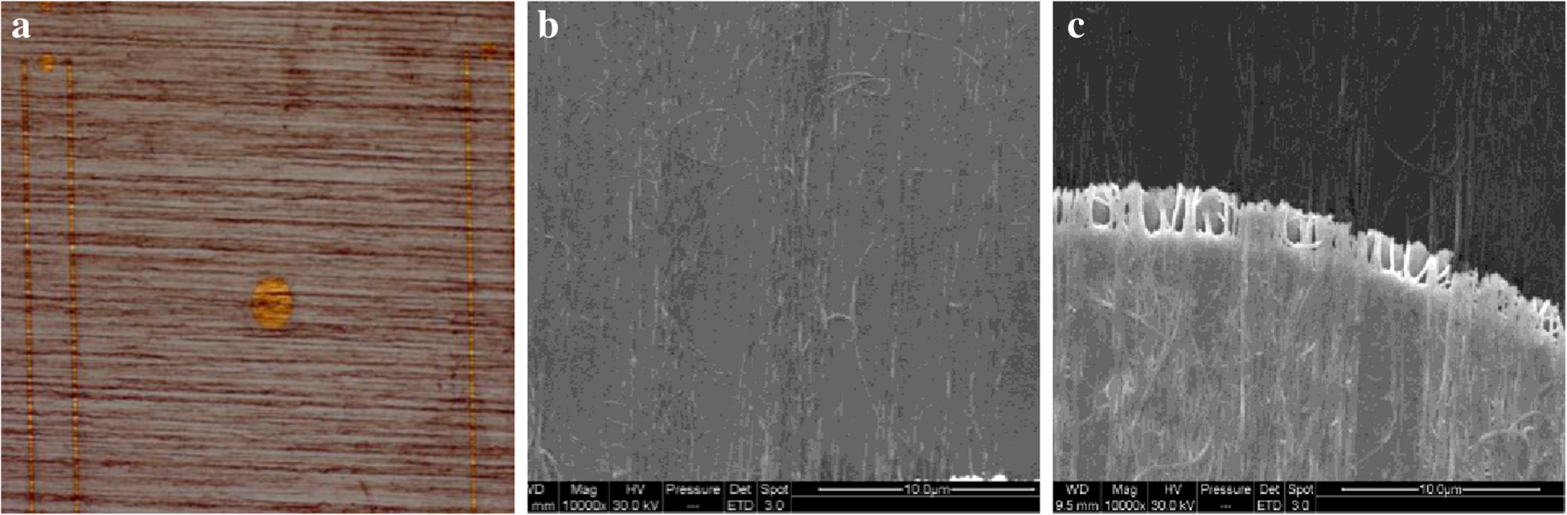
Microscope image of SACNT on LED surface (a) and relative SEM images (b) and (c).

Figure 2a showed the microscope image of the SACNTs on the LED surface at 100 times magnification. It can be seen that the SACNTs were distributed uniformly on the surface in bunches. The dark color part on the surface indicated lots of SACNTs grouped together, while the light color part had high transparency. Figure 2b,c showed the scanning electron microscopy (SEM) images of Figure 2a. SACNTs that covered the surface are quasi-aligned and lapped together forming a conducting network, which was essential for carrier transportation. Figure 2c showed the morphology of the SACNT thin film coated with 2-nm-thick Au. From our previous study, the conductivity of SACNT films can be increased obviously through the coating of Au metal, which guaranteed the current injection from the electrode to the SACNTs.

**Figure 3:**
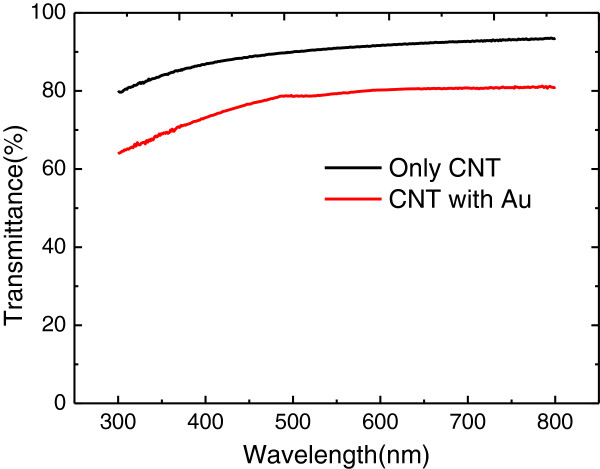
Optical transmittance measurement of SACNT and Au-coated SACNT thin film on polished glass substrate.

Figure 3 showed the optical transmittance curves of SACNT and Au-coated SACNT thin film on a polished glass substrate from 300 to 800 nm. At the wavelength of 630 nm, the optical transmittance of SACNT and Au-coated SACNT thin film was 92% and 80%, respectively. Both optical transmittance curves decreased relatively fast at wavelength below 500 nm, which indicated the SACNTs were suitable for AlGaInP LEDs at the wavelength range from 560 to 650 nm. However, the optical transmittance and the sheet resistance for SACNTs, which are two important factors for current spreading, are competing. The sheet resistance of SACNTs in this work, measured by four-probe method, was about 1,000 Ω due to the relatively high tube-tube junction resistance. While the sheet resistance of Au-coated SACNTs decreased to 130 Ω due to the high conductivity of the metal which could avoid the typically high tube-tube junction resistance. Because the Au coating was fabricated before SACNTs were put on the surface of the LED devices, it was uniformly coated on both sides of the SACNT thin films. Thereby, the contact resistance between the SACNT thin film and GaP window layer was also much decreased by the Au film.

**Figure 4:**
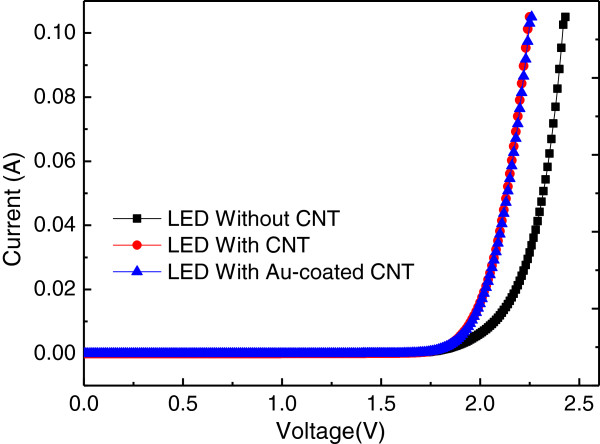
***I*-*V *** **characteristics of AlGaInP LEDs with SACNTs, Au-coated SACNTs, and without SACNTs for comparison, respectively.**

Figure 4 showed the *I*-*V* characteristics of AlGaInP LEDs with SACNTs, Au-coated SACNTs, and without SACNTs, respectively. The rectifying curves with the reverse leakage current at the level of 10^−9^ A were presented for all the samples. The forward voltage at the current injection of 20 mA was 2.02, 2.03, and 2.18 V for LEDs with SACNTs, Au-coated SACNTs, and without SACNTs, respectively. The forward voltage of LEDs with SACNTs and Au-coated SACNTs decreased a lot compared with that of bare LEDs. The work function of SACNT is about 4.7 to 5.0 eV, while for Au, it is about 5.1 to 5.5 eV. The addition of SACNT had little effect on the forward voltage in the view of work function. The decrease of forward voltage, we believe, was due to the effective current spreading, which was the same reason for UV-LED with graphene network on Ag nanowires [13]. The SACNTs and Au-coated SACNTs could spread the carriers laterally and injected the current into the junction through the top p-GaP, which could decrease the current crowding under the electrode and then better thermal performance.

**Figure 5:**
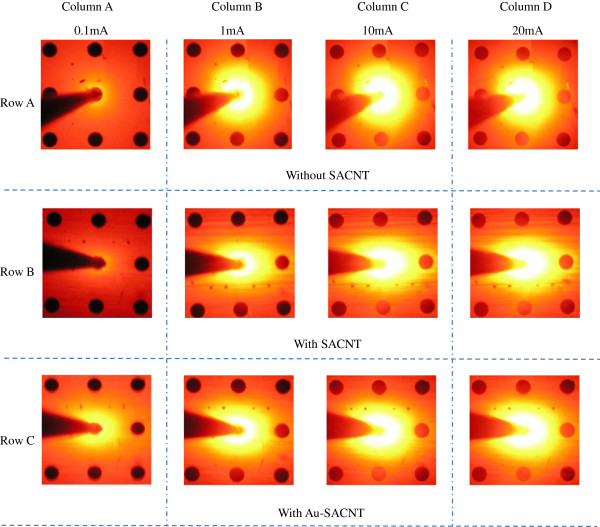
**Microscope images of LED lighting at 0.1, 1, 10, and 20 mA.** Images of LED lighting before the chip separation under the probe station taken by digital camera under the microscope for columns A, B, C, and D, in which rows A, B, and C were without and with SACNT and with Au-SACNT, respectively.

Figure 5 showed the microscope images of the three types of LED wafer before dicing under the current injection at 0.1, 1, 10, and 20 mA under the probe station taken by digital camera for columns A, B, C, and D, in which rows A, B, and C were without and with SACNT and with Au-SACNT, respectively. From column A, it was obvious to see that the whole wafer was light up with red light even at 0.1 mA. The light emission localized at the edge of the p-electrode for LED chip without SACNT. And the light-emission pattern for Au-SACNT LED was larger than that of SACNT LED. Additionally, with increasing current injection, the light-emission pattern exhibited a little difference. For SACNT LED, the ellipse spot around the probe was caused by the carrier transportation along the SACNT direction, which was the direct proof of the current-spreading effect enhanced by the SACNT. Compared with the SACNT LED, the ratio of short and long axes of the ellipse pattern of the Au-SACNT LED was smaller due to the lower sheet resistivity. The carrier transportation perpendicular to the SACNT direction was better than that of SACNT LED.

**Figure 6:**
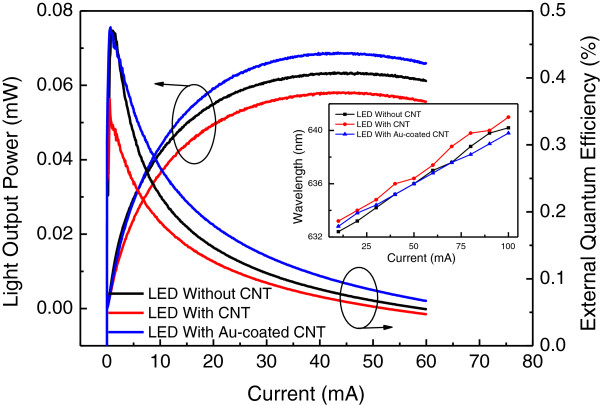
The optical output power and its external quantum efficiency dependence on the current injection.

Figure 6 illustrated the optical output power and its external quantum efficiency dependence on the current injection. The optical output power level was almost the same for the LEDs with Au-coated SACNTs and without SACNTs when the current injection is below 10 mA. After that point, the optical output power for LEDs with Au-coated SACNT increased faster. Correspondingly, the maximum external quantum efficiency of the LEDs with Au-coated SACNT and without SACNT was the same with the value of 0.46% at current injection of 0.8 mA. After that point, the external quantum efficiency decreased fast, known as efficiency droop which was studied a lot in GaN-based LED. However, the external quantum efficiency of the LEDs with Au-coated SACNT was still a little bit higher than that of LEDs without SACNT due to the current spreading. The optical output power at current injection of 20 mA for LEDs with Au-coated SACNT was improved about 9.6% and 19% compared with LEDs without and with SACNT thin film. The 10% optical power difference between the LEDs with and without SACNT was consistent with the optical transmittance measurement results.

The inset of Figure 6 showed the measured peak wavelength shift with the current injection. The peak wavelength for LEDs with SACNT, Au-coated SACNT, and without SACNT was 634, 633.8, and 633.2 nm at 20 mA, respectively. Correspondingly, the wavelength red shift was 7.8, 7, and 7.8 nm from 10 to 100 mA, respectively, which indicated better thermal performance for LEDs with Au-coated SACNT due to the relatively effective current spreading.

The improvement of optical output power for LEDs with Au-coated SACNT thin film was due to the sheet resistance competition with the p-GaP, although there existed about 20% optical transmittance loss. According to the estimation, the sheet resistance of p-GaP in this experiment is about 300 to 500 Ω. When the Au-coated SACNT thin film was put on the p-GaP, lots of carriers could spread outside the opaque metal electrode, which could have the possibility to contribute to the optical output power. The 2-nm-thick Au coating on the SACNTs could form the Au nanowire which may induce an interacting electromagnetic field with multiple quantum wells (MQWs). However, this interaction is a near-field effect. Considering the distance between of Au nanowire and quantum wells in this experiment, output enhancement due to the surface plasmon resonance can be ignored. So further decreasing the sheet resistance and improvement the optical transmittance of the current-spreading layer of SACNT thin film could increase the optical output power.

## Conclusions

The SACNT as current-spreading layer on AlGaInP LEDs was demonstrated. The voltage bias at 20 mA decreased at 0.15 V for LEDs with Au-coated SACNT, and the optical power increased about 10% compared with LEDs without SACNT due to the relatively effective current spreading. Based on the mature SACNT fabrication technique and optical transmittance performance, it is expected that SACNT could be utilized as a current-spreading layer for AlGaInP LEDs with wavelength regions from 560 to 650 nm.

## Competing interests

The authors declare that they have no competing interests.

## Authors’ contributions

GCW carried out most of the experimental work including all the measurements. GX and GCW drafted the manuscript. JYH prepared the CNT film and metal deposition. GCW and CY carried out the fabrication of LED devices. GX conducted the experiment design and analysis of all the experiments. LQQ and FSS participated in all the discussion on this study. All authors read and approved the final manuscript.
